# First record of the complete mitochondrial genome of *Botyodes diniasalis* (Walker, 1859) (Lepidoptera: Crambidae)

**DOI:** 10.1080/23802359.2023.2292745

**Published:** 2023-12-18

**Authors:** Xuan Dai, Long-Fei Deng, Xin Xiao, Xiao-Jun Yu, Jun-Xian Lv, Hong-Ying Xiong, Li Ma, Qi Chen, Lian-Yong Yang, Xing Wang

**Affiliations:** a Hunan Provincial Key Laboratory for Biology and Control of Plant Diseases and Insect Pests, Hunan Agricultural University, Changsha, China; b Hanshou County Agriculture and Rural Bureau, Changde, China; c Changde Academy of Agricultural and Forestry Sciences, Changde, China; d Tropical Biodiversity and Bioresource Utilization Laboratory, College of Science, Qiongtai Normal University, Haikou, China

**Keywords:** *Botyodes diniasalis*, margaroniini, mitogenome, phylogenetic analysis, Illumina sequencing

## Abstract

We performed the first sequencing of the complete mitogenome of *Botyodes diniasalis* by high-throughput sequencing. A circular DNA molecule of 15,219 bp in size, encoding 2 rRNAs, 22 tRNAs, and 13 PCGs, contains a non-coding AT-rich region. The overall nucleotide composition of the genome is A (39.5%), T (41.3%), C (11.3%), and G (7.8%). Phylogenetic analysis based on mitogenomic data suggest that the species *B. diniasalis* has a close evolutionary relationship with *B. principalis* in Margaroniini. The complete mitogenome of *B. diniasalis* will serve as a valuable resource for future studies on evolution, taxonomy, genetic conservation, and utilization of *Botyodes*.

## Introduction

The *Botyodes diniasalis* (Walker 1859) is an agricultural pest that causes serious damage to Poplar trees with larvae damaging Poplar foliage by leaf roll erosion. In China, it is widely distributed in Henan, Shandong, Hebei, Shanxi, Beijing, Jiangsu, Anhui, Hubei and Hunan provinces (Wu and Fang 2003). It constantly produces filaments that wrap several adjacent leaves together and the larva feed between them. It was observed on poplar trees planted in the Hanshou Vegetable Base that the larvae prefers to feed the young leaves at the top of the seedlings, which can feed up the young leaves in 3-5 days when they occur, forming bald tips and seriously affecting the natural growth of the plantlet. Nevertheless, for this agriculturally critical pest, most investigations have concentrated on exploring its migration (Song et al. 2021), and tests of defense to the chemical elements (Lin et al. 2020). Recent studies based on molecular and morphological data classified it as a Margaroniini (Mally et al. 2019), and further showed a sister relationship with *Botyodes principalis* (Matsui et al. 2022). The mitogenome of *B. principalis* is sequenced (GenBank accession number: MZ823351). However, the complete mitogenome of *B. diniasalis* is not reported in NCBI and other databases. Therefore, we firstly sequenced and annotated the complete mitogenome of *B. diniasalis*. Subsequent phylogenetic reconstruction helped to verify the phylogenetic position of this species.

## Materials

**Figure 1.**
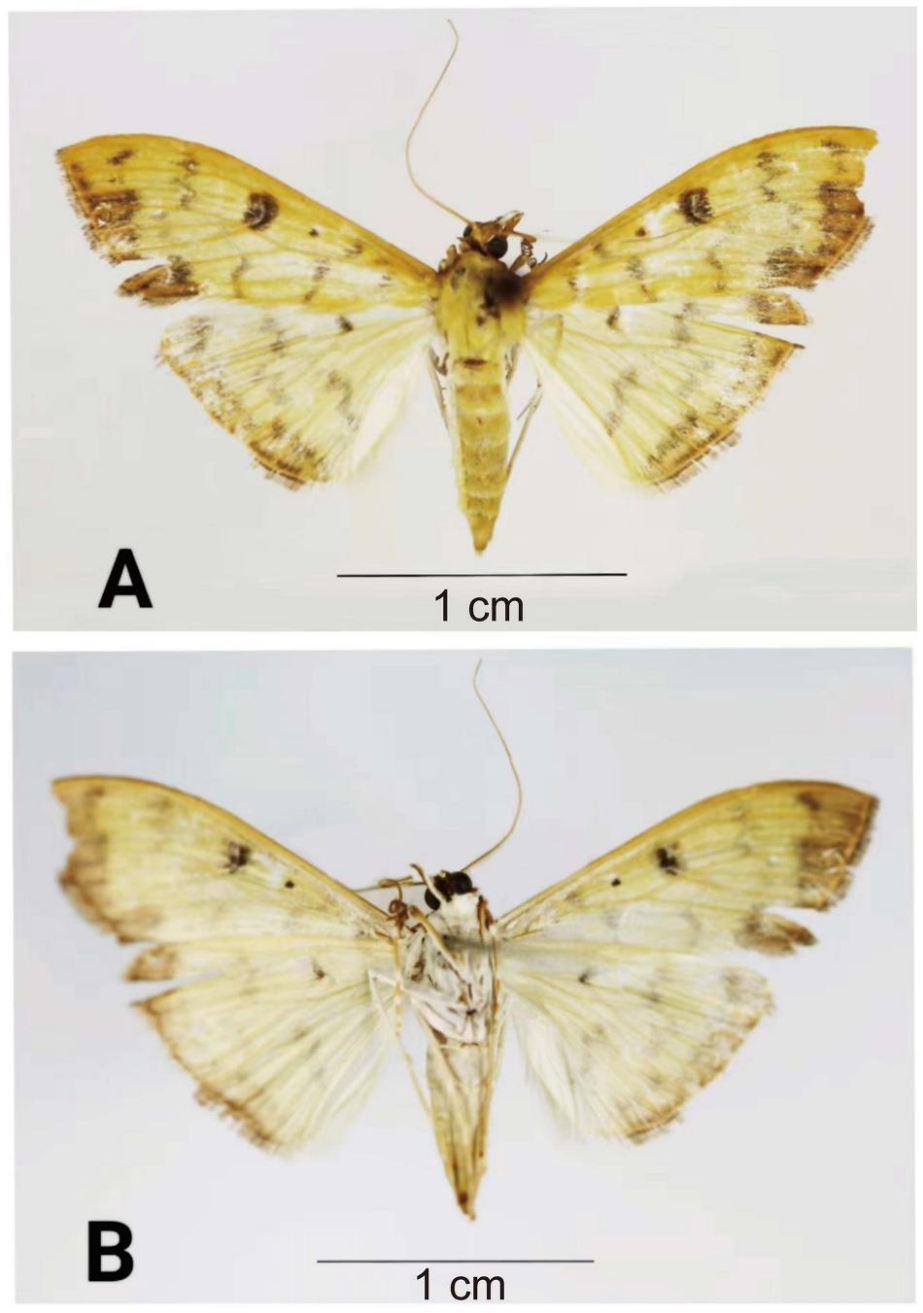
Reference images of adult of *Botyodes diniasalis*. (A) the dorsal view of *B. diniasalis*; (B) the ventral view of *B. diniasalis*. These images from our specimen were taken by DX.

*B. diniasalis* has the following morphological characteristics. The adult wingspan is about 30 mm. It is yellow, rather slender and white beneath. The wings have brownish lines and the forewings have ferruginous marginal band and brown marks. The hindwings are ferruginous at the tips. The adults of *B. diniasalis* (Figure 1), identified according to Walker (1859), were obtained in dead state by Xuan Dai using ultraviolet light traps method on 30^th^ of September 2022 at the Hanshou National Vegetable Base (Changde, China, 28.91°N, 111.95°E). The studied insect specimens and genomic DNA are kept at the Insect Herbarium of Hunan Agricultural University, Changsha, China (voucher code: HAUHL081593, contact person: Guo-Hua Huang, ghhuang@hunau.edu.cn).

## Methods

Genomic DNA was isolated using whole body tissues of *B. diniasalis* following the protocol provided in the SteadyPure Universal Genomic DNA Extraction Kit Ver.1.0 (Changsha, China). The Illumina Novaseq 6000 platform of Berry Genomics (Beijing, China) was employed to carry out 2 × 150 bp paired-end sequencing. The fastp was used to filter the raw reads (Chen et al. 2018). The complete mitogenome of *B. diniasalis* was assembled from scratch using NOVOPlasty (Dierckxsens et al. 2017) and GetOrganelle (Jin et al. 2020). The MITOS (Bernt et al. 2013) was used to annotate the *B. diniasalis* mitogenome. The tRNA scan-SE (Lowe and Chan 2016) was used to predict the tRNA structure. Final adjustments were made using Geneious (Kearse et al. 2012). To identify and draw the sequence and direction of genes, we used MitoZ v3.6 (Meng et al. 2019), and for genomic visualization, we utilized the Circos module (Krzywinski et al. 2009).

To establish the phylogenetic context of the reported mitogenome, we downloaded 17 published mitogenomes of Margaroniini closest to *B. diniasalis* from NCBI as ingroups and selected 5 published mitogenomes of Spilomelinae as the outgroups. The amino acid sequences of 13 PCGs in their mitogenomes were manually extracted using Geneious, then aligned using MAFFT (Katoh and Standley 2013) and concatenated using FASConCAT-g (Kück and Longo 2014). The optimal partitioning strategy and substitution models were selected by PartitionFinder (Lanfear et al. 2017). A phylogenetic analysis with maximum likelihood (ML) was then carried out using the IQ-TREE (Minh et al. 2020). At last, FigTree 1.4.4 (https://github.com/rambaut/figtree/) was used to display the resulting phylogenetic tree.

## Results

**Figure 2.**
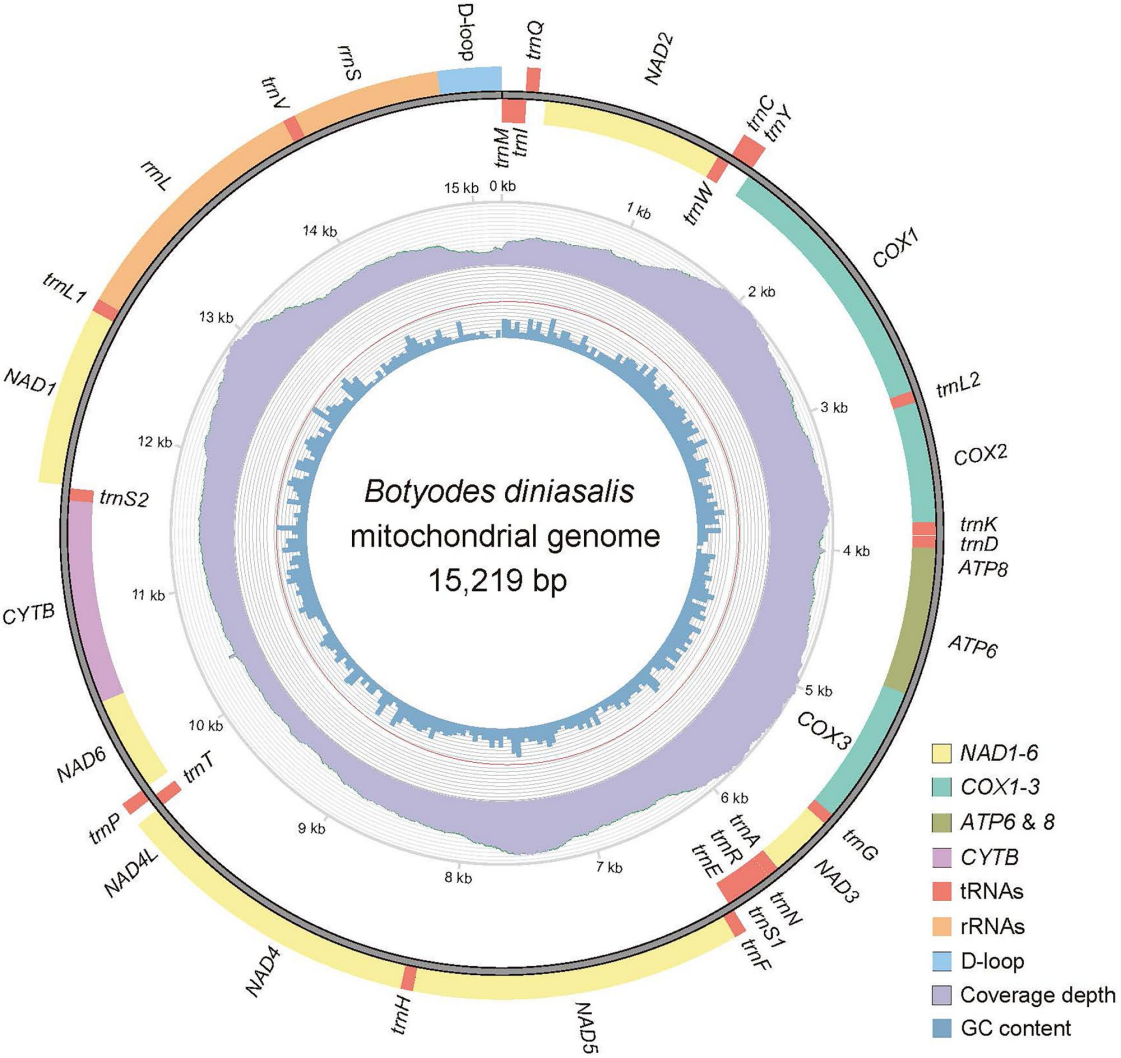
Mitogenome pattern map of *Botyodes diniasalis*. Genes inside the black circle are coded in the minority strand (N-strand); genes outside the black circle are coded in the minority strand (J-strand). the inner and outer colors are represented: the innermost layer represents GC content, the Middle layer represents coverage depth, and the outermost layer represents genes.

**Figure 3.**
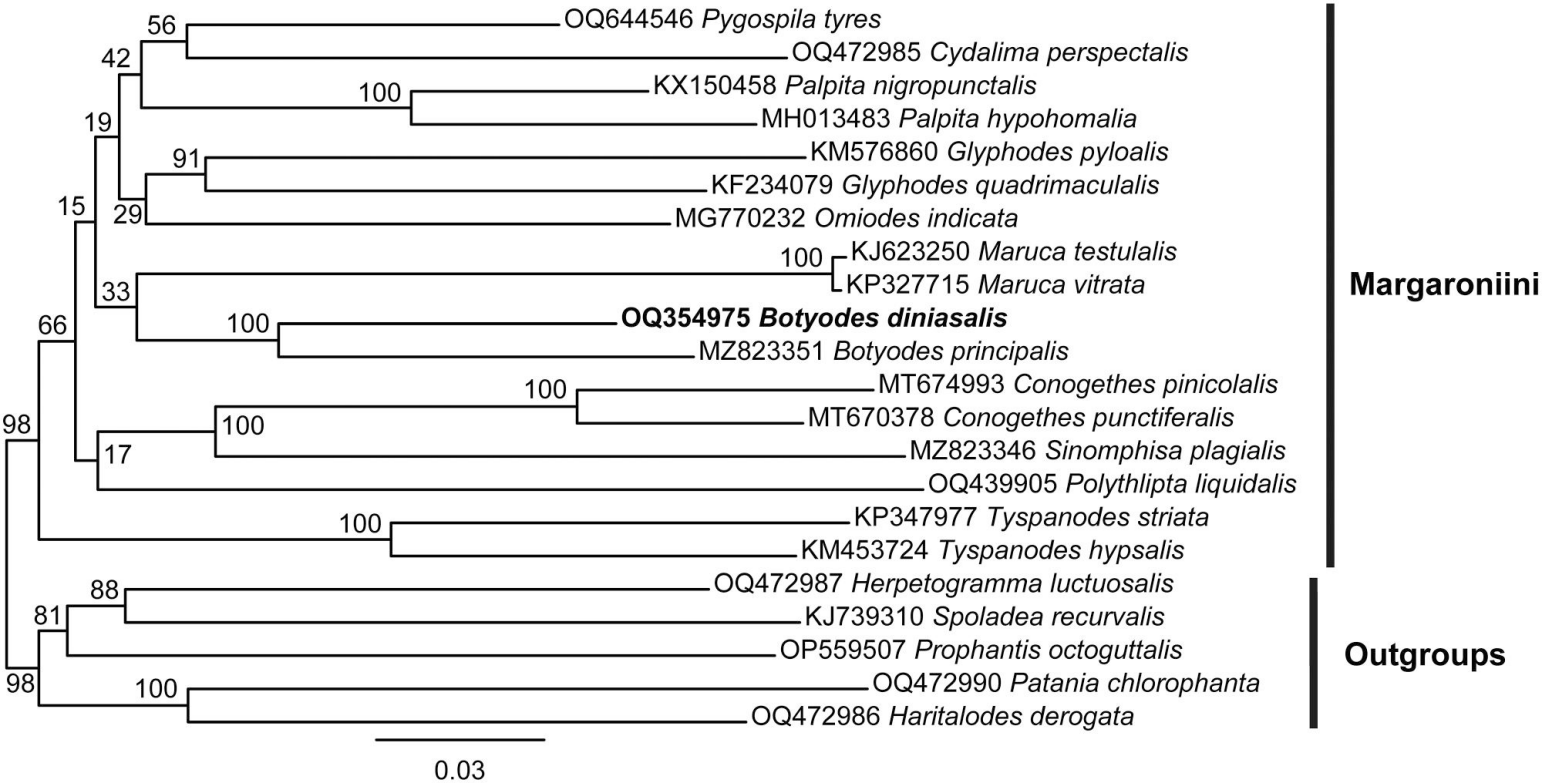
Maximum likelihood (ML) tree of 17 species within the tribe Margaroniini based on 13 PCGs of the mitogenome with 5 Spilomelinae species as outgroups. The following sequences were used: OQ644546 *Pygospila tyres* (Kalawate et al. 2022), OQ472985 *Cydalima perspectalis* (Gao et al. 2021), KX150458 *Palpita nigropunctalis* (Chen 2022), MH013483 *Palpita hypohomalia* (Yang et al. 2018a), KM576860 *Glyphodes pyloalis* (Kong and Yang 2016), KF234079 *Glyphodes quadrimaculalis* (Park et al. 2015), MG770232 *Omiodes indicata* (Yang et al. 2018b), KJ623250 *Maruca testulalis* (Zou et al. 2016), KP327715 *Maruca vitrata* (Margam et al. 2011), MZ823351 *Botyodes principalis* (Liu et al. 2021), MT674993 *Conogethes pinicolalis* (Ra et al. 2021), MT670378 *Conogethes punctiferalis* (Ra et al. 2021), MZ823346 Sinomphisa plagialis (Liu et al. 2022), OQ439905 *Polythlipta liquidalis* (Yang et al. 2023), KP347977 *Tyspanodes striata* (Ma et al. 2016), KM453724 *Tyspanodes hypsalis* (Wang et al. 2016), OQ472987 *Herpetogramma luctuosalis* (Zhou and Yang 2023a), KJ739310 *Spoladea recurvalis* (He et al. 2015), OP559507 *Prophantis octoguttalis* (Tang and Du 2023), OQ472990 *Patania chlorophanta* (Zhou and Yang 2023b), OQ472986 *Haritalodes derogata* (Zhao et al. 2016). The numbers on the nodes refer to the bootstrap value.

The complete mitogenome of *B. diniasalis* is a circular DNA molecule with a length of 15219 bp (GenBank accession number: OQ354975), consisting of 13 PCGs, 22 tRNA genes, 2 rRNA genes and an AT-rich region. Twenty-two genes are transcribed in the J strand and fifteen in the N strand. The nucleotide composition is A (39.5%), T (41.3%), C (11.3%), and G (7.8%), and the AT nucleotide content is 80.8% (Figure 2). With the exception of *COX1*, which has a CGA start codon, the remaining 13 PCGS have an ATN start codon, while TAA and T serve as termination codons. The 22 tRNAs ranged in length from 64 bp (*trnR*) to 71 bp (*trnK*). The length of *rrnS* is 778 bp, while the length of *rrnL* is 1346 bp. The intergenic regions span 108 bp, between 18 pairs of adjacent genes, and the length is 2 bp ∼ 46 bp. There are 23 bp of overlapping nucleotides scattered between 12 pairs of adjacent genes, and the length is 1 bp ∼ 7 bp. The A + T-rich region between 12S rRNA and *trnM* is 336 bp long. A molecular phylogenetic tree is shown in Figure 3 which is based on 13 PCGs concatenated sequence of 22 species, and showed that the species *B. diniasalis* has a close evolutionary relationship with *B. principalis* in Margaroniini.

## Discussion and conclusion

The first mitogenome sequence of *B. diniasalis* is reported in this study. Based on gene content, AT content, and gene order, the newly sequenced mitogenome shows a close resemblance to *B. principalis* (Accession number: MZ823351) within the tribe Margaroniini. Moreover, it has similarity to those of other Margaroniini mitogenomes (Liu et al. 2021), which illustrated that the mitogenomes of Margaroniini could be conserved in structures and characteristics. Phylogenetic inference based on 13 PCGs from 22 mitochondrial genomes indicates a relatively clear position of *B. diniasalis* in Margaroniini. Taken together, the complete mitogenome of *B. diniasalis* can contribute to the understanding of the mitogenome characteristics and determination of the phylogenetic position of the tribe Margaroniini.

## Supplementary Material

Supplemental Material

## Data Availability

The genome sequence data that support the findings of this study are openly available in GenBank of NCBI under the accession number OQ354975. The associated BioProject, SRA, and Bio-Sample numbers are PRJNA929694, SRR25319645, and SAMN32968917, respectively.
