# Supplementary figures and images for: First record of the complete mitochondrial genome of Botyodes diniasalis (Walker, 1859) (Lepidoptera: Crambidae)
Source: Mitochondrial DNA B Resour. 2023 Dec 18;8(12):1401–5. doi: 10.1080/23802359.2023.2292745 (PMC10732176; doi:10.1080/23802359.2023.2292745)

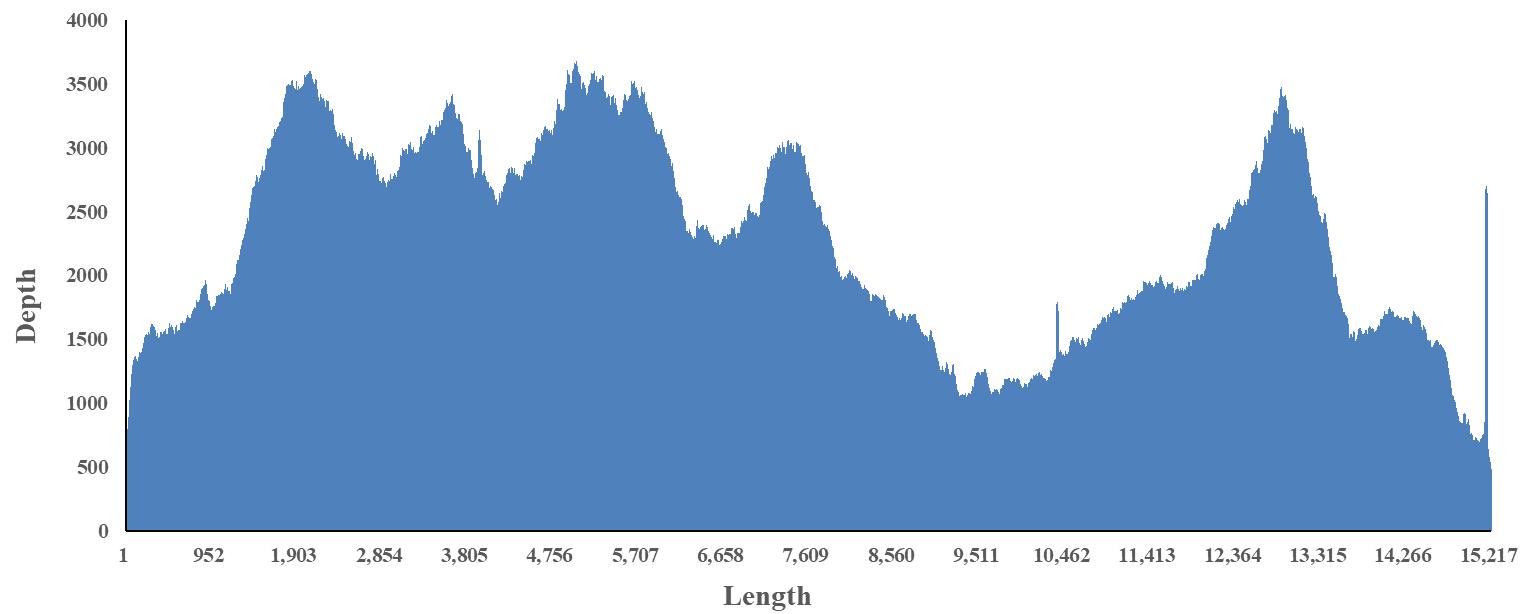

Supplement: Supplemental Material [file TMDN_A_2292745_SM1185.jpg]
